# Establishment of Parentage Identification Method for Sea Urchin *Strongylocentrotus intermedius* Based on SSR-seq Technology

**DOI:** 10.3390/genes15050630

**Published:** 2024-05-16

**Authors:** Xuechun Jiang, Lei Liu, Hao Guo, Peng Liu, Wenzhuo Tian, Fanjiang Ou, Jun Ding, Weijie Zhang, Yaqing Chang

**Affiliations:** Key Laboratory of Mariculture & Stock Enhancement in North China’s Sea, Ministry of Agriculture and Rural Affairs, Dalian Ocean University, Dalian 116023, China; 15395747873@163.com (X.J.); yqchang@dlou.edu.cn (Y.C.)

**Keywords:** *Strongylocentrotus intermedius*, parentage identification, microsatellite

## Abstract

To establish a parentage identification method for *Strongylocentrotus intermedius*, 15 microsatellite loci and simple sequence repeat sequencing (SSR-seq) technology were used to perform SSR sequencing and typing of the validation population with known pedigree information and the simulation population. Cervus v3.0 was used for gene frequency statistics, simulated analysis, and parentage identification analysis. The results showed that, in validation population, using 15 microsatellite loci, the highest success rate of parent pairs identification was 86%, the highest success rate of female parent identification was 93%, and the highest success rate of male parent identification was 90%. The simulated population was analyzed using 12–15 loci, and the identification rate was up to 90%. In cases where accurate parentage was not achieved, individuals could exhibit genetic similarities with 1–3 male or female parents. Individuals identified as lacking a genetic relationship can be selected as parents to prevent inbreeding. This study shows that parent pairs or single parents of most offspring can be identified successfully using these 15 selected loci. The results lay a foundation for the establishment of a parentage identification method for *S. intermedius*.

## 1. Introduction

*Strongylocentrotus intermedius* has been the most important cultured sea urchin species in China since its introduction in 1989 [1]. Owing to limitations associated with water temperature, food, and other culturing conditions, the culturing areas of *S. intermedius* are mainly distributed in cold-water areas, such as Dalian, Yantai, and Weihai, in the North China sea area [1]. In recent years, the demand for sea urchins in China’s domestic market has increased annually, promoting the rapid development of the sea urchin aquaculture industry. However, with culturing scale expansions and culturing environment deterioration, slow growth and frequent disease have increasingly become prominent challenges [2]. At present, although China has bred two novel sea urchin varieties (*S. intermedius* “Dajin” and “Fengbao No. 1”) [2], they are yet to meet the growing demand in the sea urchin aquaculture industry. More new sea urchin varieties urgently need to be cultivated.

Individual marking is an important part of plant and animal breeding processes. The ideal markers are physical tags, such as fluorescent markers injected intramuscularly into fish and shrimp, which allow rapid, high-throughput identification of individuals, and parentage tracing, which is very convenient for recording the phenotypic values of traits and estimating breeding values for individuals [3]. In sea urchins, early studies of population or individual markers used external tags such as smearing sieve plates, inserting metal tag strips, positioning tags, or external plastic tags [4,5,6,7]. Chemical labels (tetracycline labeling, fluorescent dyes, and fluorescent elastomers) have also been used to label sea urchins [8,9] but have serious limitations for uniquely identifying individuals or large numbers of families. Recent developments in passive integrated transponder (PIT) tags [10] allow for the use of internal devices to mark sea urchins individually. However, PIT tags and external plastic tags are cumbersome with regard to operation and manual design, in addition to having limited reliability [11]. Due to the difficulty of individual labeling, in the selective breeding process of sea urchins, different families must be reared in separate tanks, which confound the common environmental effects caused by separate tank culturing with the genetic effects of families, thereby decreasing the accuracy of breeding value estimation [4]. In this technical context, the breeding researchers hope to use molecular markers to identify genetic relationships, facilitating mixed-pool cultivation. This approach will significantly reduce the human and material costs while enhancing the accuracy of breeding value estimations.

Microsatellite markers with simple sequence repeat (SSR) technology are among the most commonly used molecular markers in genetic diversity analysis in animal and plant populations. They have advantages such as high polymorphism, good stability, easy availability, co-dominant inheritance, and adherence to Mendelian genetic laws. In recent years, SSR markers have been used extensively for the genetic breeding of aquatic animals such as fish, crustaceans, and shellfish [12,13]. At present, parentage identification of aquatic animals based on microsatellite technology has achieved good results for many species. To assess the feasibility of the genetic identification of the cultured population of *Fenneropenaeus chinensis*, Dong et al. [14] used 5 microsatellite loci and achieved a success rate of 92.9%. Yang et al. [15] used 10 microsatellite loci to identify 5 full-sib families of *Siniperca chuatsi*, and the results showed that 95% of the offspring could be identified correctly. In addition, Zhu et al. [16] selected 14 microsatellite markers to evaluate the parent-child relationship of *Penaeus monodon* and achieved a 99% identification accuracy. The primary methods for microsatellite marker (SSR) typing include high-concentration agarose gel electrophoresis, polyacrylamide gel electrophoresis with silver staining (PAGE-silver stain), fluorescent capillary electrophoresis, and SSR-seq technology [17]. Agarose gel electrophoresis is less commonly used for direct SSR typing due to its low resolution, difficulty in distinguishing closely-spaced alleles, and susceptibility to human error [17]. PAGE is complex and labor intensive, reducing the efficiency of large-scale molecular marker analysis and often resulting in issues such as uneven bands, deep backgrounds, gel tears, and bubbles [17]. Capillary electrophoresis has been the most utilized SSR typing method in recent years. However, it also has some technical shortcomings such as amplification artifacts, imprecise sizing, length homoplasy, and limited multiplex capability [18]. SSR-seq is an innovative microsatellite typing technology that integrates multiplex PCR with next-generation sequencing (NGS). High-depth SSR-seq (1000–5000×) can directly detect the sequence information of different alleles at the same SSR loci, achieving single-base resolution, and can also accurately indicate the number of repetitions of SSR repeat units for each allele [19,20]. Compared to paternity testing based on NGS technology and SNP typing, SSR-seq is more cost effective and does not require complex bioinformatics analysis or specialized hardware [21].

This study used 15 selected microsatellite loci and SSR-seq technology to perform sequencing, typing, and parent–child identification analyses on 100 *S. intermedius* individuals from 10 full-sib families, as well as 50 individuals from a mixed fertilization population, with the aim of achieving high identification accuracy and establishing a parent–child identification system for *S. intermedius*. After the establishment of this identification system, it is expected to achieve the mixed culturing of families in the breeding of *S. intermedius*. This will help reduce the impact of common environmental effects on breeding value estimation and also help reduce the cultivation costs in the family breeding process.

## 2. Materials and Methods

### 2.1. Experimental Material

A total of 32 individuals of *S. intermedius* were selected for parentage identification from a breeding family population (8th generation family breeding population) cultivated in the Key Laboratory of Mariculture and Stock Enhancement in North China’s Sea, Ministry of Agriculture and Rural Affairs, Dalian Ocean University, as candidate parents. These candidate parents were induced to spawn individually by injection of potassium chloride solution (0.5 M) in a volume of 1.5 mL for each parent. During the spawning induction process, 9 sea urchins failed to spawn, so a total of 23 parent individuals (12 males and 11 females) participated in the mating. The sex of parents is determined by the production of male and female gametes. According to the genealogical relationship, it was confirmed that there was no genetic relationship between these 23 parents. Sperm from 1 male and eggs from 1 female were randomly chosen for fertilization to produce a full-sib family. Using this method, 10 full-sib families were established as a validation population (marked as Y). Embryos from each family were hatched independently, and larvae were also bred independently after hatching. During breeding, each family’s tank was clearly marked to prevent any mixing of offspring between families. In addition, eggs from 4 female sea urchins were randomly selected to be mixed in equal proportions, and sperm from 10 male sea urchins was selected to be mixed in equal proportions, after which the sperm and egg were mix-fertilized and placed into a tank for hatching and rearing. Thus, a population in which the parents of the offspring were unknown was constructed to serve as a simulated population (marked as H). The parent compositions of the validation population and the simulated population are shown in Table 1.

At 6 months old, 10 individuals from each of the full-sib families in the validation population were randomly selected for paternity testing, totaling 100 individuals. Simultaneously, 50 individuals from the simulated population were chosen randomly for paternity testing.

### 2.2. Extraction of Genomic DNA

Genomic DNA was extracted from 23 parents and 150 offspring from tube feet using a marine animal tissue genomic DNA extraction kit (Qiagen Biochemical Technology Co., Ltd., Beijing, China), and DNA integrity was detected using 1% agarose gel electrophoresis. The concentration and purity of the DNA were determined using a nucleic acid protein analyzer (Biochrom, Cambridge, UK). The DNA samples were stored in a refrigerator at −80 °C.

### 2.3. Screening of Microsatellite Loci

Our previous study [22] identified 15 highly polymorphic SSR loci, and the screening process can be summarized as follows: A total of 75 microsatellite loci were selected from the literature, and their primers were synthesized by Shanghai Bioengineering Biological Co., Ltd. (Shanghai, China). The 75 SSR loci were screened using polyacrylamide gel electrophoresis, yielding 21 polymorphic loci. Additionally, 41 microsatellite loci were identified from the transcriptome database of *S. intermedius* [23,24], for which primers were designed and synthesized. The polymorphic microsatellite data and newly designed site information were submitted to Shanghai Tianhao Biotechnology Co., Ltd. (Shanghai, China) for typing feasibility verification. Primers were screened using a gradient PCR reaction. The PCR mixture was composed of 25 μL, including 12.5 μL of 2× Accurate Taq premix (Ai Kerui Bioengineering Co., Ltd., Shanghai, China), 1 μL of template, 1 μL each of forward and reverse primers, and 10.5 μL of ddH_2_O. The PCR amplification included: pre-denaturation at 94 °C for 5 min, denaturation at 98 °C for 10 s, a gradient annealing cycle (the annealing temperature was the average annealing temperature of the forward and reverse primers, −2, −1, ±0, +1, +2, and +3, respectively, for a total of 6 temperatures) for 30 s, extension at 72 °C for 40 s for 5 cycles per temperature, totaling 30 cycles, followed by a final extension at 72 °C for 10 min and storage at 4 °C. PCR products were analyzed using 8% non-denaturing polyacrylamide gel electrophoresis. Following screening and validation, 25 primers displaying clear bands and good diversity were selected, and a total of 15 loci with 4 or more alleles were selected for SSR typing [22]. Information for the primers of the 15 loci is presented in Table 2.

### 2.4. SSR-seq Typing Process

Using 15 pairs of primers for each panel as standards, the optimized primers were mixed as a multiplex touchdown PCR panel. The standard genome was used as a template for amplification to optimize a multiplex touchdown PCR panel system. The PCR mixture was composed of 10 μL, including 1 μL of 10× buffer (Takara, Dalian, China), 0.07 μL of 5 U/μL HotStart Taq (Takara, Dalian, China), 1.2 μL of 2.5 mM dNTP, 0.6 μL of 25 mM MgCl_2_, 1 μL of template, 0.5 μL each of forward and reverse primers, and 5.13 μL of ddH_2_O. The PCR amplification included: Pre-denaturation at 95 °C for 2 min, 11 cycles with denaturation at 95 °C for 20 s, an initial annealing temperature of 63 °C for 40 s, and extension at 72 °C for 1 min. The annealing temperature was then reduced 0.5 °C each cycle over the touchdown cycles, followed by an additional 24 cycles at the annealing temperature of 65 °C for 30 s, totaling 35 cycles, followed by a final extension at 72 °C for 2 min and storage at 4 °C. This method was used to amplify the target fragment of the 23 parents and 150 offspring involved in the experiment, and the products of the same individual were mixed to ensure that the amount of primers amplified at each site was equivalent. Subsequently, a specific tag sequence compatible with the Illumina platform was introduced at the end of the library by PCR amplification using primers containing the index sequence (index primer). The PCR mixture was composed of 10 μL, including 1 μL of 10× buffer (Takara, Shanghai, China), 0.04 μL of 5 U/μL HotStart Taq (Takara, Shanghai, China), 0.8 μL of 2.5 mM dNTP, 0.2 μL of 25 mM MgCl_2_, 1 μL of template, 0.5 μL each of forward and reverse index primers, and 5.96 μL of ddH_2_O. The PCR amplification included: Pre-denaturation at 95 °C for 2 min, 12 cycles with denaturation at 95 °C for 20 s, annealing temperature of 60 °C for 40 s, and extension at 72 °C for 1 min. This was followed by a final extension at 72 °C for 2 min and storage at 4 °C. The index PCR amplification products of all samples were mixed in equal amounts, and the final FastTarget^TM^ sequencing library was obtained by tapping and recycling. The fragment length distribution of the library was verified using an Agilent 2100 bioanalyzer (Agilent Technologies, Santa Clara, CA, USA). After accurate quantification of the molar concentration of the library, high-throughput sequencing was performed on the Illumina HiSeq platform in the 2 × 150/2 × 250 double-end sequencing mode. Based on the sequencing results, the number of repetitions of the repetitive units at each locus was determined for each individual, thereby determining the genotypes of 15 loci for each individual.

### 2.5. Data Analysis

Using the geom_density tool in ggplot, a density distribution map was constructed based on the number of reads counted. Based on the genotyping results, Cervus v3.0 (http://www.fieldgenetics.com/pages/aboutCervus_New.jsp, accessed on 1 January 2022) was used for gene frequency statistics, simulated analysis, and parentage identification analysis. The exclusion rate of 15 loci was calculated, and the number of loci was reduced gradually according to their polymorphic information content (PIC). A total of 100 individuals from the validation population were subjected to biparental sex-known parent pair identification, uniparental known male parent analysis, and female parent analysis. As the parents of the 100 individuals selected in the validation population were known, their data can validate the accuracy of molecular markers for parentage identification. If the molecular identification results align with the physical markers (tank records), the identification is deemed correct; discrepancies were treated as identification errors. The formulae for calculating the actual identification rate of parent pairs (RPV), male parent (RMV), and female parent (RFV) of the validation population are as follows:

$$\mathrm{RPV}=\frac{\mathrm{Number}\;\mathrm{of}\;\mathrm{individuals}\;\mathrm{whose}\;\mathrm{male}\;\mathrm{and}\;\mathrm{female}\;\mathrm{parents}\;\mathrm{identified}\;\mathrm{correctly}}{100}$$

$$\mathrm{RMV}=\frac{\mathrm{Number}\;\mathrm{of}\;\mathrm{individuals}\;\mathrm{whose}\;\mathrm{male}\;\mathrm{parent}\;\mathrm{identified}\;\mathrm{correctly}}{100}$$

$$\mathrm{RFV}=\frac{\mathrm{Number}\;\mathrm{of}\;\mathrm{individuals}\;\mathrm{whose}\;\mathrm{female}\;\mathrm{parent}\;\mathrm{identified}\;\mathrm{correctly}}{100}$$

Based on the cumulative identification rate of different numbers of loci and the actual identification rate of the validation population, it is possible to determine the number of loci that can achieve the highest identification accuracy for the simulated population. For the identification of 50 individuals within the simulated population, the identification results of 12 and more than 12 loci were selected. When the number of loci involved in identification was 12–15, the same parent pair was identified and was considered accurate. If the parent pairs identified by 13–15 loci were the same, but different from the one identified by 12 loci, the identification of 13–15 loci was considered an accurate parent. If the parent pairs identified by 14–15 loci were the same, but different from the one identified by 12 or 13 loci, then the parent identification result based on 14–15 loci was considered accurate. If the identification results of 15 loci differed from those of 14 loci, the test was considered to have failed to accurately identify the real parent. Then, parents identified based on 12–15 loci were all possible parents. The formula for calculating the actual identification rate of the simulated population (RS) is as follows:

$$\mathrm{RS}=\frac{\mathrm{Accurately}\;\mathrm{identify}\;\mathrm{the}\;\mathrm{number}\;\mathrm{of}\;\mathrm{individuals}\;\mathrm{of}\;\mathrm{real}\;\mathrm{parents}}{50}$$

We used IBM SPSS Statistics (IBM Corp., Armonk, NY, USA) for correlation analysis of the polymorphic information content (PIC) of loci and the exclusion rate of the first parent (E-1P) when parental genotypes were unknown.

**Figure 1:**
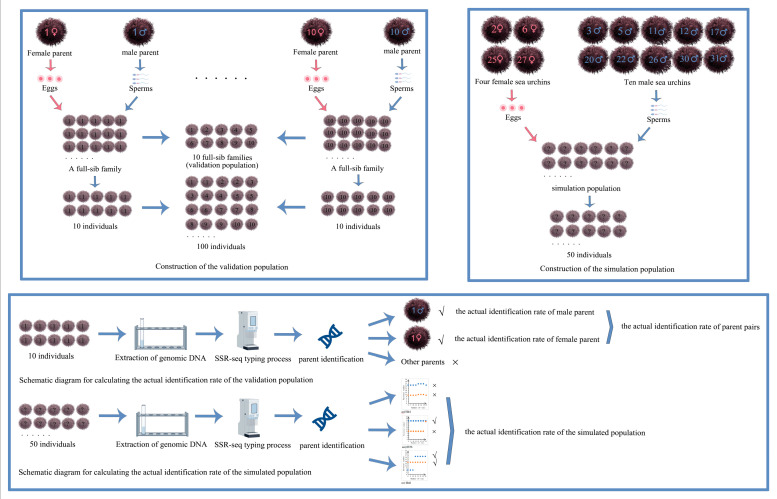
Experimental process of parentage identification. Note: ? marked on an individual indicates that its parents are unknown; √ means parent identification succeed; × means parent identification failed.

Figure 1 shows the experimental process of parentage identification.

## 3. Results

### 3.1. Sequencing Results

After comparing the sequencing data with the reference genome using BLAST^+^, the enrichment efficiency of the target fragments was calculated. The enrichment efficiency of the parent sample is shown in Table 3, and that of the offspring samples is listed in Table 4.

### 3.2. SSR-seq Typing Results

**Figure 2:**
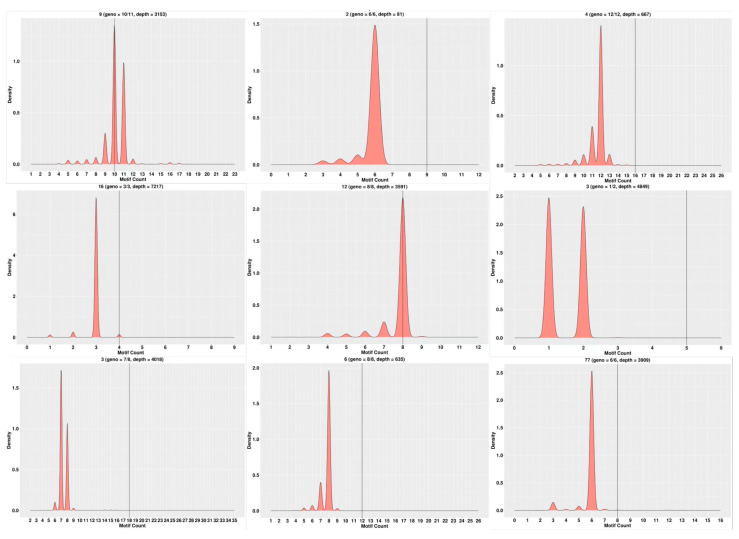
Examples of partial SSR loci typing in some parents. Note: The horizontal coordinates represent the number of motifs, the vertical coordinates represent density, and the black vertical line in the middle represents the number of motifs in the reference sequence.

**Figure 3:**
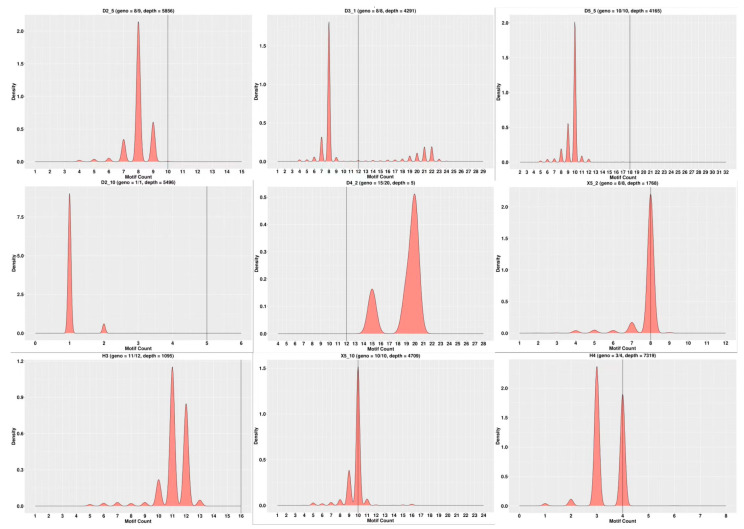
Examples of partial SSR loci typing in some offspring. Note: The horizontal coordinates represent the number of motifs, the vertical coordinates represent density, and the black vertical line in the middle represents the number of motifs in the reference sequence.

Using the sequencing data, the typing of 23 parents and 15 loci per parent was completed. Examples of partial SSR loci typing in some parents are shown in Figure 2, and examples of partial SSR loci typing in some offspring are shown in Figure 3.

### 3.3. Cumulative Relationship Analysis between Exclusion Rate and Number of Loci

**Figure 4:**
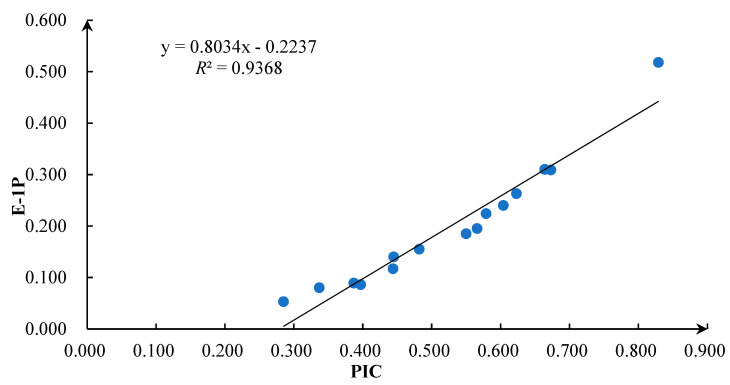
Linear relationship between site polymorphic information content (PIC) and first parent exclusion rate (E-1P). Note: blue dots means 15 microsatellite loci.

Correlation analysis between PIC and the exclusion rate of the first parent (E-1P) revealed a significant correlation (*R*^2^ = 0.9368, *p* < 0.01) (Figure 4).

Parent–child identification in 10 offspring families of *S. intermedius* was carried out using Cervus v3.0. The confidence interval was 95%, and 15 microsatellite markers were accumulated in descending order of PIC size. The exclusion rate of the first parent (E-1P) was 0.053–0.518, and the cumulative exclusion rate of the first parent (CE-1P) was 0.969 493 (Table 5). When the number of loci used reached 10, CE-1P could reach more than 95%, the exclusion rate of the second parent (E-2P) was 0.159–0.686, and the cumulative exclusion rate of the second parent (CE-2P) was 0.998 867. When the number of sites used was 4, a CE-2P greater than 95% could be achieved. When the number of sites used was 12, a CE-2P greater than 99.73% could be achieved, the parental exclusion rate (E-PP) was 0.266–0.856, and the cumulative exclusion rate of parents (CE-PP) was 0.999 991. When the number of used sites was 5, a CE-PP greater than 99.73% could be achieved. In total, 9 of the 15 loci deviated from the Hardy–Weinberg equilibrium, accounting for 60%.

### 3.4. Verification of the Identification Results of the Population

Sea urchins can visually determine sex after spawning. Therefore, three identification methods—known parental sex, maternal analysis, and paternal analysis—were used (Table 6). When the sex of both parents is known, the simulated identification rate of parental pair can reach 100%. When 7 loci were used, the simulated identification rate in the female parent analysis achieved 100%, and when 6 loci were used in the male parent analysis, the simulated identification rate achieved 100%. Using 15 loci, the actual identification rate of parent pairs was 86%, the actual identification rate of female parent analysis was 93%, and the actual identification rate of male parent analysis was 88%.

### 3.5. Identification Results of Simulated Population

**Figure 5:**
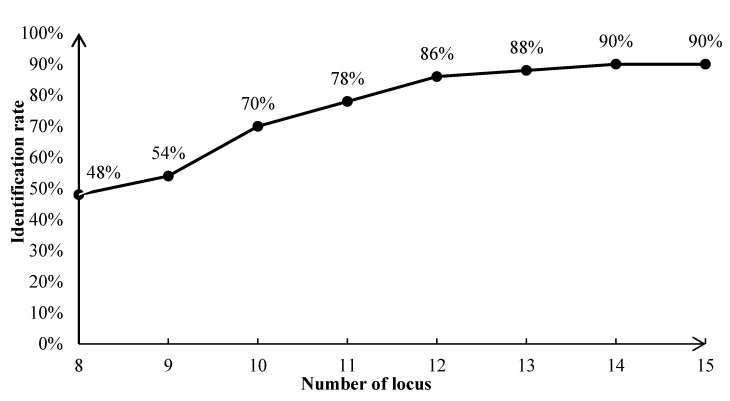
Parent identification rates of 50 individuals in simulated population.

According to the number of loci from more to less, firstly, the simulation analysis of parents’ sex known was carried out. When the number of loci was 8, a simulation identification rate of 100% could be achieved. Therefore, the optimal number of loci for parent–child identification was 8–15. Parentage was determined based on the magnitude of the likelihood ratio value (LOD). If LOD < 0, the candidate parent is definitely not the genetic parent of the offspring; if LOD = 0, the candidate parent and other parents in the population have the same probability of being the true parent of the offspring; if LOD > 0, there is a high probability that the candidate parent is the true parent of the offspring. The larger the LOD value, the closer the candidate parent is to the true parent [25]. The parents with positive LOD values were counted in the identification results, as shown in Supplementary Figure S1. The identification of 50 offspring at different numbers of loci is shown in Figure 5.

**Figure 6:**
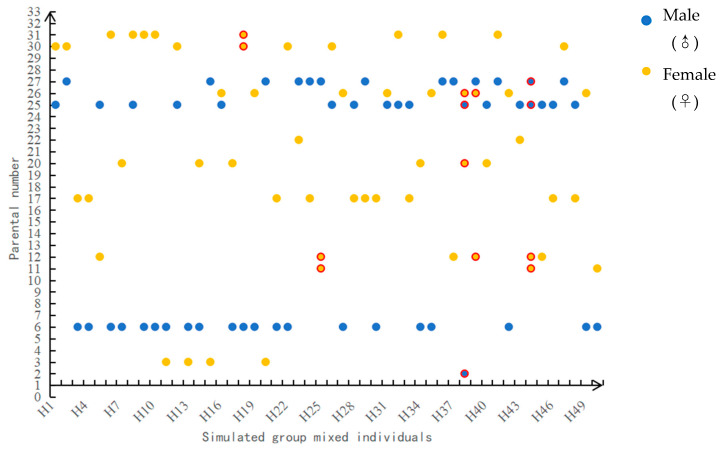
Identification of 50 individuals mixed in a simulated population. Note: The parents marked in red circles indicate that a unique parent has not been identified.

When the number of loci involved in the identification was 10, a CE-1P of 95.24% could be achieved, which is more than 95% and increases the probability that the identified parent is the actual parent [26]. Based on the identification results of the validation population, with an increase in the number of loci, identification success rate increased gradually. When the number of loci involved in the identification was 10, an actual identification rate of 85% could be achieved. However, when the number of loci was 11, the actual identification rate decreased to 84%. Increasing the number of loci to 12 brought the identification rate back to 85% and stabilized it. Therefore, the identification results for 12 and more than 12 loci in the simulated population were considered. Consequently, the identification rate and results of the simulated population in the present study are illustrated in Figure 5 and Figure 6, and the highest identification rate that could be achieved was 90%. Five individuals (H18, H25, H38, H39, and H44) had uncertain parentage, three individuals (H18, H25, and H39) successfully matched the female parent, and two individuals (H38 and H44) showed potential relationships with to 2–3 male parents or female parents.

### 3.6. Contribution Rate of Different Parents to Offspring Population

The validation population in the present study was full-sib families in which the contribution rate of parents to the offspring was known, whereas the contribution rate of parents to the offspring in the simulated population was unknown. By counting the individuals who successfully identified unique parental pairs in the simulated population, the parental identification rates are shown in Table 7.

Among the 45 mixed progeny populations that successfully identified the only parent pair, 3 female parents contributed to the offspring population, and the contribution rate of female parents ranged from 22.22% to 44.44%. Among them, the contribution rate of No. 6 female parent was the highest, while the No. 27 female parent was the lowest. The No. 2 female parent did not participate in reproduction. Nine male parents contributed to the offspring, and the contribution rate of male parents ranged from 2.22% to 22.22%. Among them, the contribution rate of No. 17 male parent was the highest, that of No. 11 male parent was the lowest, and No. 5 male parent was not involved in reproduction.

## 4. Discussion

### 4.1. Cumulative Relationship Analysis between Exclusion Rate and Number of Loci

The PIC of the locus was significantly positively correlated with the exclusion rate of the first parent, indicating that when the number of loci was the same, the more abundant the polymorphism of the locus, and the higher the accuracy of parentage identification. In addition, the higher the polymorphism of the microsatellite loci, the higher the accuracy of parent identification. Using microsatellite loci with high polymorphism can greatly improve the efficiency of parent–child identification [27]. Therefore, it is speculated that it is necessary to select loci with higher polymorphism to achieve higher identification accuracy with fewer microsatellite loci. In the parentage identification analysis, the exclusion rate of microsatellite markers is the simplest and most effective dataset that enables the identification of the pedigree relationship [28]. In addition, according to the conclusion by Vankan and Faddy [26], when CEP ≥ 99.73%, a parent–child relationship can be confirmed; when 99% < CEP ≤ 99.73%, there is a high likelihood of a parent–child relationship; when 95% < CEP ≤ 99%, there may be parent–child relationship; when CEP < 80%, there is no parent–child relationship. In the present study, when the parental genotype was unknown and the number of loci used was 10, there could be a parent–child relationship between the candidate parents and the offspring. When the genotype of the single parent was known, the number of loci used reaches 4 to determine that there may be a parent–child relationship between the candidate parent and the offspring; when the number of loci used was 12, the parent–child relationship between the candidate parent and offspring could be confirmed. When the parent genotype was known, the parent-child relationship between the candidate parents and offspring can be confirmed using 5 loci. The loci in this study can basically satisfy the parentage identification of the sea urchin *S. intermedius*. When selecting the loci for parentage identification, the polymorphism level of the loci, the technology used for typing and reproductive design (whether the number of parents and sex are known), and other information need to be considered as they may influence the success rate of parentage identification [27]. However, parentage identification should be based on a small number of loci, low research costs, and high identification efficiency. Among the 15 loci in the present study, 9 loci deviated from the Hardy–Weinberg equilibrium in the offspring population, and the frequency of invalid alleles at 5 loci was >10%. Multiple loci deviated from the Hardy–Weinberg equilibrium. One possible reason for this phenomenon is that there are more inbred individuals in the population, and the population may decompose into a series of family groups with closer genetic relationships or inbreeding. Heterozygosity due to inbreeding can lead to segregation of genes in the offspring and a tendency for the genetic composition of the offspring population to be pure, with most of the lack of heterozygote (excess of homozygote) due to null alleles, ultimately leading to loci that deviate from the Hardy–Weinberg equilibrium [29]. This result is consistent with the reality of the offspring population in this study.

### 4.2. Analysis of Parental Contribution Rate

The contribution rate of parents to their offspring reflects the role of natural selection to a certain extent, which is a popular topic in reproductive biology and evolutionary ecology [30]. The unbalanced contribution rate of parents to offspring may lead to allele loss. After repeated generations of reproduction, the level of genetic diversity in the population is reduced [31]. This imbalance also discards lethal genes or bad traits in the population. 

In the present experiment, the parents of 45 individuals were accurately identified, and only three female parents participated in reproduction, all of which had high contribution rates. The egg quality provided by the three female parents should be good. However, one female parent did not participate in reproduction, but it was reported as a potential parent of the H39. The possible reasons may be related to the quality of the eggs produced by the maternal parent, with eggs that were not fertilized or died during incomplete development in the larval stage after fertilization. This may also be related to the small number of high-quality eggs the parent provides and the fewer offspring, whose offspring individuals were not selected in the random sample [32]. Notably, the contribution rate of all male parents varied significantly, as evidenced by the contribution rate of No. 5 male parent being 0 and the contribution rate of No. 17 male parent being 22.22%. The total contribution rate of 17, 20, 26, 30, and 31 in male parents was 77.78%, which was much higher than that of other male parents, indicating that the sperm involved in fertilization and fertilization success mainly came from the five male parents. This phenomenon may be related to the low fertilization and survival rates of some parents [33]. However, the accuracy of parentage identification had a direct impact on the contribution rate. Sekino et al. [34] used four microsatellite molecular markers to identify the parentage of Japanese flounder (*Paralichthys olivaceus*) and observed that the contribution rates between the parents were very different. In the present study, the identification rate was 90%, and five individuals were not identified as parents; therefore, there was also a certain impact on the contribution rate of the parents. To maintain balance for unknown reasons, increasing the number of breeding parents is one solution, and it is also necessary to develop microsatellite loci with higher polymorphism that can be added to the parentage identification system to further improve its accuracy [27].

## 5. Conclusions

In the present study, 15 loci were used to conduct a preliminary study on the parentage identification of selective breeding populations with known parents and mixed crosses with unknown parents. Under the premise that the sex of both parents was known, parent pair analysis, male parent analysis, and female parent analysis all had good identification success rates; a 90% success rate of the simulated population could be achieved under the premise that the parental sex of the simulated population was known. Based on the current method, parentage identification of sea urchin populations can be carried out effectively, providing a reference for the development of a more accurate parentage identification system and theoretical support for future production and breeding to avoid inbreeding.

## Figures and Tables

**Table 1:** Parent compositions of validation population (Y) and simulated population (H).

Offspring Populations	Female Parent Number	Male Parent Number
Y1	7	17
Y2	8	12
Y3	32	31
Y4	24	12
Y5	25	17
Y6	23	18
Y7	13	20
Y8	21	18
Y9	6	20
Y10	27	28
H	2	3
	6	5
	25	11
	27	12
	—	17
	—	20
	—	22
	—	26
	—	30
	—	31

**Table 2:** 15 pairs of SSR-seq primer information.

Number	Locus	Repeat Motif	Amplified Fragment Size/bp	Primer Sequence (5′-3′)
1	SSR1	(CT)_12_	196~226	F: TCGTCATGAGATGGTCGCT R: CATTTTACCGTGGTGGGGTC
2	SSR2	(AG)_13_	179~187	F: CGCAGGATGCAGTGATACC R: ATTCCACCAGTATCCCAGCT
3	SSR3	(CT_)18_	136~180	F: GCGCTTAATCTTTGGATAATTG R: CTGTAGTCGCTCCGCATGT
4	SSR4	(AG)_12_	181~219	F: GGGAAGTTTTCCCCACTGAC R: TGTCCATAACGCCACATTCG
5	SSR7	(AC)_10_	199~213	F: TCCCATATGATTGCTCGTGC R: AGCATTCACCGCGAAACTG
6	SSR14	(AG)_10_	165~179	F: ATCCCAAACTACGTTCAACC R: GGCTGCCTAGTTGCATAAAT
7	SSR16	(CT)_16_	146~246	F: CCTTGGAATGAGAACTTGT R: ACCGATTTTACTTGACCTG
8	SSR17	(AT)_6_	231~237	F: CTGTTTGGATGAGTGGAAT R: TTTGAACGAGCTTGCCTT
9	SSR18	(TTGACT)_4_	112~130	F: GTCAGGTAGCTATATGTTC R: TGGTGATAAACATGTCAGAA
10	SSR19	(GCA)_8_	93~102	F: AGCTCCTAGGGTTCTTACC R: ACATGGGTGGAGAGGTG
11	SSR20	(GAA)_5_	147~156	F: CTAATAGCCCTATGCCGCGT R: ATACACCACACGATTCGCAC
12	SSRA6	(TGA)_6_	179~194	F: AAGCAGCCATTAAGGAAATG R: CAAGCAGGTTATCCGTTTCA
13	SSRA9	(TC)_9_	185~211	F: AAGCGAGCTTATGTCTAGTA R: CTAGAACCTTCATCAACTCT
14	SSRA10	(AC)_6_	148~168	F: CACGTATTTCGGATGGTGAC R: CTTATTATTAGCGCACGTCAT
15	SSRA22	(TCTG)_6_	186~202	F: GAAGAACCATGGACTTACTACA R: TGTTGTGAGAAAGGTAGCG

**Table 3:** Statistics of enrichment efficiency of parental samples.

	Clean Reads	Raw Reads	Clean Reads Ratio	Q20	Q30
Average of all the parents	52,480	57,020	0.92	100.00%	99.99%

**Table 4:** Statistics of enrichment efficiency of progeny samples.

	Clean Reads	Raw Reads	Clean Reads Ratio	Q20	Q30
Average of all sampled offspring	45,295	53,518	0.85	100.00%	99.98%

**Table 5:** Cumulative exclusion rates for 15 microsatellite sites.

Locus	Exclusion Rate of the First Parent E-1P	Exclusion Rate of the Second Parent E-2P	Parental Exclusion Rate E-PP	Cumulative Exclusion Rate of the First Parent CE-1P	Cumulative Exclusion Rate of the Second Parent CE-2P	Cumulative Exclusion Rate of Parents CE-PP	Hardy-Weinberg Equilibrium HWE	Ineffective Allele Frequency
								*F* (null)
SSR1	0.518	0.686	0.856	—	—	—	ns	0.004 5
SSR7	0.309	0.482	0.671	0.666938	0.837348	0.952624	**	−0.055 1
SSR4	0.310	0.494	0.696	0.770187	0.917698	0.985598	**	0.266 5
SSR14	0.263	0.435	0.623	0.830628	0.953499	0.994570	ns	0.039 6
SSRA6	0.240	0.418	0.615	0.871277	0.972937	0.997910	**	0.410 2
SSR3	0.224	0.393	0.574	0.900111	0.983573	0.999110	ns	0.011 4
SSRA10	0.195	0.349	0.518	0.919589	0.989306	0.999571	ns	0.026 6
SSRA22	0.185	0.330	0.481	0.934465	0.992835	0.999777	**	0.362 6
SSR20	0.155	0.292	0.444	0.944623	0.994927	0.999876	ns	0.058 6
SSR18	0.140	0.250	0.378	0.952376	0.996195	0.999923	**	0.302 6
SSR17	0.117	0.257	0.405	0.957948	0.997173	0.999954	*	−0.086 5
SSRA9	0.086	0.223	0.369	0.961565	0.997804	0.999971	ns	−0.012 7
SSR16	0.089	0.222	0.359	0.964985	0.998291	0.999981	ns	−0.077 1
SSR2	0.080	0.212	0.351	0.967786	0.998653	0.999988	*	0.177 0
SSR19	0.053	0.159	0.266	0.969494	0.998868	0.999991	ns	−0.034 8

Notes: ns. In accordance with Hardy-Weinberg equilibrium; *. Significant deviation from Hardy-Weinberg equilibrium (*p* < 0.05); **. Significant deviation from Hardy-Weinberg equilibrium (*p* < 0.01).

**Table 6:** Relationship between identification rates of offspring and the number of loci.

Number of Locus	Analog Identification Rate			Actual Identification Rate		
	Parents Analysis	Female Parent Analysis	Male Parent Analysis	Parents Analysis	Female Parent Analysis	Male Parent Analysis
1	0.00%	3.00%	5.00%	2.00%	9.00%	0.00%
2	1.00%	10.00%	20.00%	7.00%	30.00%	26.00%
3	2.00%	26.00%	23.00%	23.00%	30.00%	47.00%
4	14.00%	50.00%	76.00%	48.00%	70.00%	68.00%
5	35.00%	79.00%	99.00%	59.00%	80.00%	72.00%
6	65.00%	94.00%	100.00%	61.00%	79.00%	75.00%
7	90.00%	100.00%	100.00%	73.00%	88.00%	79.00%
8	100.00%	100.00%	100.00%	78.00%	87.00%	84.00%
9	100.00%	100.00%	100.00%	81.00%	88.00%	88.00%
10	100.00%	100.00%	100.00%	85.00%	90.00%	90.00%
11	100.00%	100.00%	100.00%	84.00%	91.00%	88.00%
12	100.00%	100.00%	100.00%	85.00%	92.00%	88.00%
13	100.00%	100.00%	100.00%	85.00%	92.00%	88.00%
14	100.00%	100.00%	100.00%	86.00%	93.00%	88.00%
15	100.00%	100.00%	100.00%	86.00%	93.00%	88.00%

**Table 7:** Contribution rates of parents to the offspring in the simulated population.

Parent Number		Number of Offspring Individuals	Contribution Rate/%
Female	2	0	0.00%
	6	20	44.44%
	25	15	33.33%
	27	10	22.22%
Male	3	4	8.89%
	5	0	0.00%
	11	1	2.22%
	12	3	6.67%
	17	10	22.22%
	20	5	11.11%
	22	2	4.44%
	26	7	15.56%
	30	6	13.33%
	31	7	15.56%

## Data Availability

Data will be made available on request.

## Supplementary Materials

The following supporting information can be downloaded at: https://www.mdpi.com/article/10.3390/genes15050630/s1, Figure S1: Identification of 50 individuals in a simulated population with different number of loci.

## References

[1] Agatsuma Y. (2013). Strongylocentrotus intermedius. Dev. Aquacult. Fisheries Sci..

[2] Lawrence J.M., Zhao C., Chang Y. (2019). Large-scale production of sea urchin (*Strongylocentrotus intermedius*) seed in a hatchery in China. Aquacult. Int..

[3] Lü H., Fu M., Zhang Z., Su S., Yao W. (2020). Marking fish with fluorochrome dyes. Rev. Fish. Sci. Aquac..

[4] Hagen N.T. (1996). Tagging sea urchins: A new technique for individual identification. Aquaculture.

[5] Agatsuma Y., Nakata A., Matsuyama K. (2000). Seasonal foraging activity of the sea urchin *Strongylocentrotus nudus* on coralline flats in Oshoro Bay in south-western Hokkaido, Japan. Fisheries Sci..

[6] Duggan R.E., Miller R.J. (2001). External and internal tags for the green sea urchin. J. Exp. Mar. Biol. Ecol..

[7] Clemente S., Hernández J.C., Brito A. (2007). An external tagging technique for the long-spined sea urchin *Diadema aff. antillarum*. J. Mar. Biol. Assoc. Uk..

[8] Ellers O., Johnson A.S. (2009). Polyfluorochrome marking slows growth only during the marking month in the green sea urchin *Strongylocentrotus droebachiensis*. Invertebr. Biol..

[9] Martinez A.S., Byrne M., Coleman R.A. (2013). Unique tagging of small echinoderms: A case study using the cushion star *Parvulastra exigua*. Methods Ecol. Evol..

[10] Gibbons W.J., Andrews K.M. (2004). PIT tagging: Simple technology at its best. Bioscience.

[11] Peterson C.H., Black R. (1994). An experimentalist’s challenge: When artifacts of intervention interact with treatments. Mar. Ecol. Prog. Ser..

[12] Fuji K., Kobayashi K., Hasegawa O., Coimbra M.R.M., Sakamoto T., Okamoto N. (2006). Identification of a single major genetic locus controlling the resistance to lymphocystis disease in Japanese flounder (*Paralichthys olivaceus*). Aquaculture.

[13] Selly S.L.C., Hickey J., Stevens J.R. (2014). A tale of two hatcheries: Assessing bias in the hatchery process for Atlantic salmon (*Salmo salar* L.). Aquaculture.

[14] Dong S., Kong J., Zhang T., Meng X., Wang R. (2006). Parentage determination of Chinese shrimp (*Fenneropenaeus chinensis*) based on microsatellite DNA markers. Aquaculture.

[15] Yang M., Tian C., Liang X., Lv L., Zheng H., Yuan Y., Zhao C., Huang W. (2014). Parentage determination of mandarin fish (*Siniperca chuatsi*) based on microsatellite DNA markers. Biochem. Syst. Ecol..

[16] Zhu K., Yu W., Huang J., Zhou F., Guo H., Zhang N., Jiang S., Zhang D. (2017). Parentage determination in black tiger shrimp (*Penaeus monodon*) based on microsatellite DNA markers. Aquac. Int..

[17] Kristamtini K., Taryono T., Basunanda P., Murti R.H. (2016). High resolution microsatellite marker analysis of some rice landraces using metaphor agarose gel electrophoresis. Indone. J. Biotechnol..

[18] Darby B.J., Erickson S.F., Hervey S.D., Ellis-Felege S.N. (2016). Digital fragment analysis of short tandem repeats by high-throughput amplicon sequencing. Ecol. Evol..

[19] Lepais O., Chancerel E., Boury C., Salin F., Manicki A., Taillebois L., Dutech C., Aissi A., Bacles C.F.E., Daverat F. (2020). Fast sequence-based microsatellite genotyping development workflo. PeerJ.

[20] Šarhanová P., Pfanzelt S., Brandt R., Himmelbach A., Blattner F.R. (2018). SSR-seq: Genotyping of microsatellites using next-generation sequencing reveals higher level of polymorphism as compared to traditional fragment size scoring. Ecol. Evol..

[21] Laoun A., Harkat S., Lafri M., Gaouar S.B.S., Belabdi I., Ciani E., Groot M.D., Blanquet V., Leroy G., Rognon X. (2020). Inference of breed structure in farm animals: Empirical comparison between SNP and microsatellite performance. Genes.

[22] Liu L., Zhang W., Liu Y., Leng X., Ou F., Qi X., Li X., Ding J., Chang Y. (2023). Evaluation of the genetic diversity and genetic structure of multiple generation selection populations and unselected common population of sea urchin (*Strongylocentrotus intermedius*) using SSR-seq. J. Fish. China.

[23] Zhang W., Lv Z., Li C., Sun Y., Jiang H., Zhao M., Zhao X., Shao Y., Chang Y. (2019). Transcriptome profiling reveals key roles of phagosome and NOD-like receptor pathway in spotting diseased *Strongylocentrotus intermedius*. Fish Shellfish Immun..

[24] Zhang W., Wang Z., Leng X., Jiang H., Liu L., Li C., Chang Y. (2019). Transcriptome sequencing reveals phagocytosis as the main immune response in the pathogen-challenged sea urchin *Strongylocentrotus intermedius*. Fish Shellfish Immun..

[25] Kalinowski S.T., Taper M.L., Marshall T.C. (2007). Revising how the computer program CERVUS accommodates genotyping error increases success in paternity assignment. Mol. Ecol..

[26] Vankan D.M., Faddy M.J. (1999). Estimations of the efficacy and reliability of paternity assignments from DNA microsatellite analysis of multiple-sire matings. Anim. Genet..

[27] Weng Z., Yang Y., Wang X., Wu L., Hua S., Zhang H., Meng Z. (2001). Parentage analysis in giant grouper (*Epinephelus lanceolatus*) using microsatellite and SNP markers from genotyping-by-sequencing data. Genes.

[28] Jones A.G., Ardren W.R. (2003). Methods of parentage analysis in natural populations. Mol. Ecol..

[29] Ma K., Feng J., Li J., Ding H. (2010). Twenty-four novel polymorphic microsatellite markers from oriental river prawn (*Macrobrachium nipponense*). Conserv. Genet. Resour..

[30] Yang W., Zheng J., Jia B., Chang S., Liu H., Li C., Wang G., Yang F. (2017). SSR marker and its research progress to animal genetics and breeding. Genom. Appl. Biol..

[31] Zhao R., Cheng Z., Lu W., Lu B. (2006). Estimating genetic diversity and sampling strategy for a wild soybean (*Glycine soja*) population based on different molecular markers. Chinese Sci. Bull..

[32] Deneke V.E., Pauli A. (2021). The fertilization enigma: How sperm and egg fuse. Annu. Rev. Cell Dev. Bi..

[33] Primmer C.R., Borge T., Lindell J., Satre G.P. (2002). Single-nucleotide polymorphism characterization in species with limited available sequence information: High nucleotide diversity revealed in the avian genome. Mol. Ecol..

[34] Sekino M., Saitoh K., Yamada T., Kumagai A., Hara M., Yamashita Y. (2003). Microsatellite-based pedigree tracing in a Japanese flounder *Paralichthys olivaceus* hatchery strain: Implications for hatchery management related to stock enhancement program. Aquaculture.

