# Supplementary material for: Establishment of Parentage Identification Method for Sea Urchin Strongylocentrotus intermedius Based on SSR-seq Technology
Source: Genes (Basel). 2024 May 16;15(5):630. doi: 10.3390/genes15050630 (PMC11120681; doi:10.3390/genes15050630)
Supplement: Supplementary file 1 [file genes-15-00630-s001.zip › genes-2968502-supplementary.pdf]

Supplementary Figure

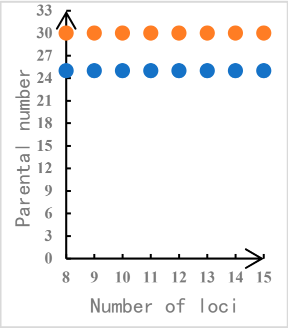

a) H1

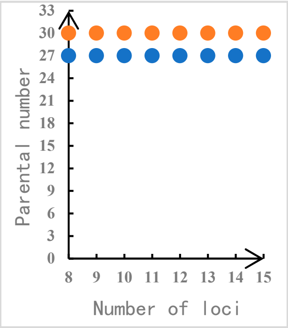

b) H2

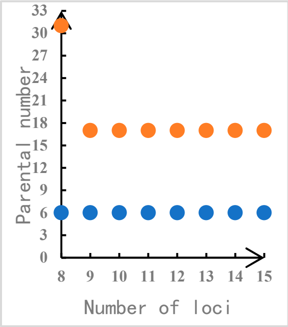

c) H3

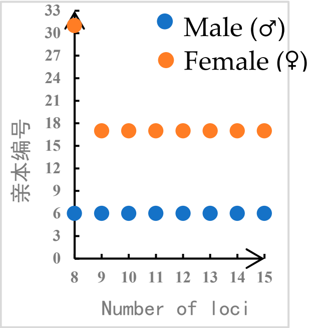

d) H4

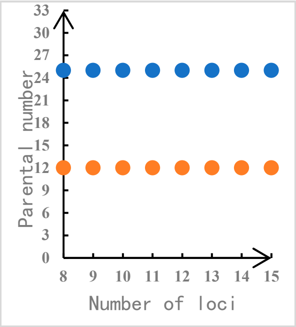

e) H5

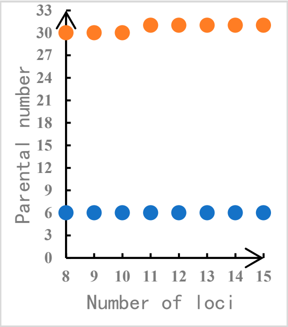

f) H6

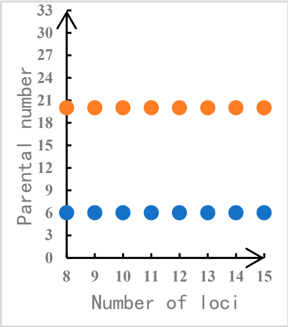

g) H7

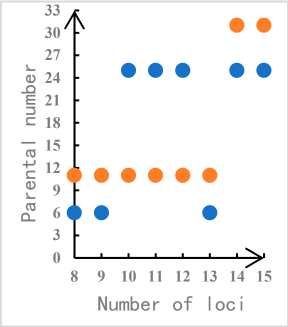

h) H8

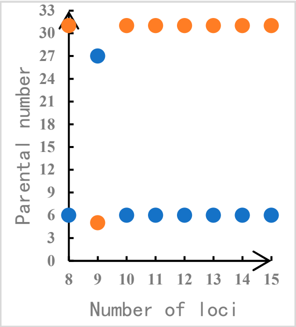

i) H9

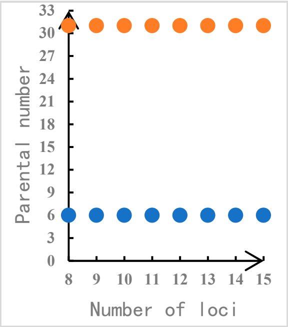

j) H10

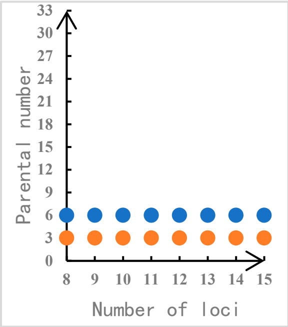

k) H11

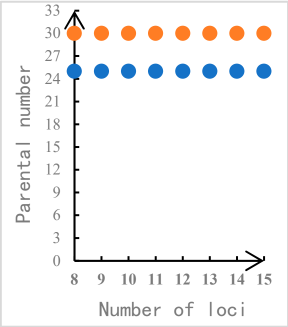

l) H12

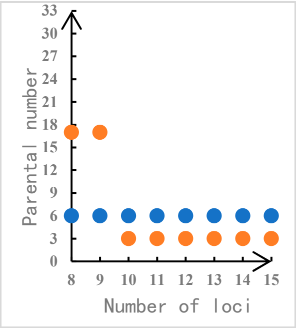

m) H13

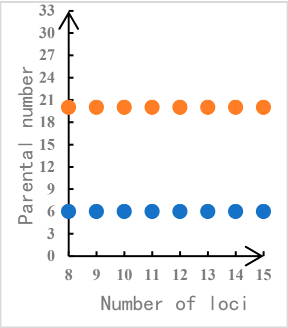

n) H14

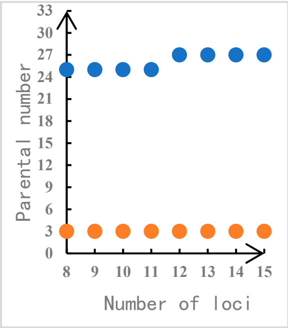

o) H15

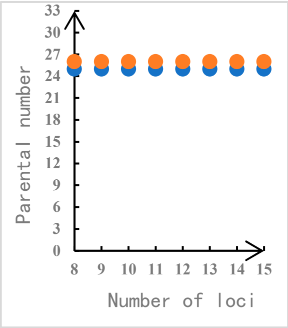

p) H16

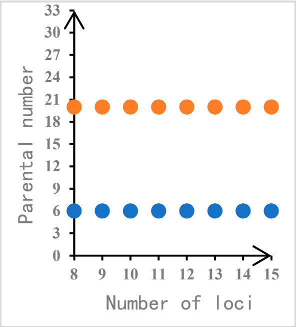

q) H17

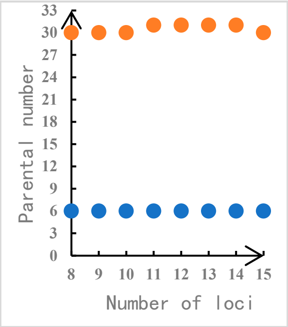

r) H18

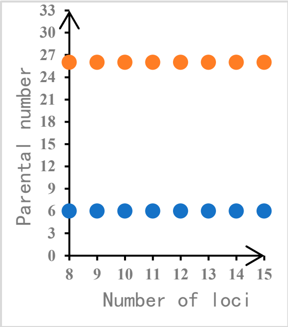

s) H19

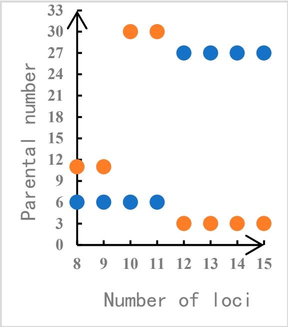

t) H20

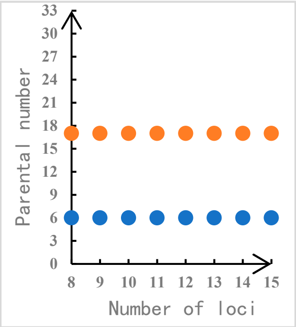

u) H21

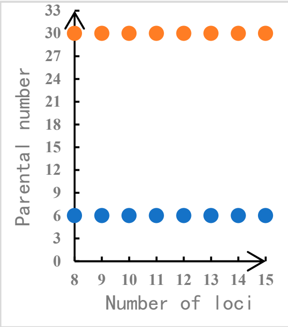

v) H22

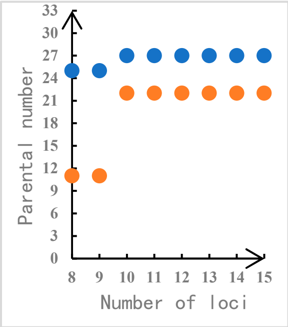

w) H23

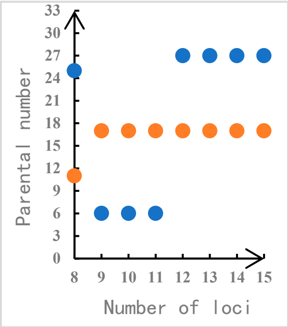

x) H24

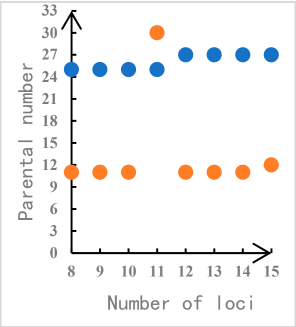

y) H25

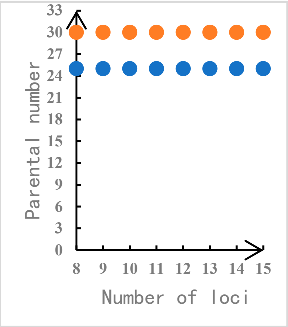

z) H26

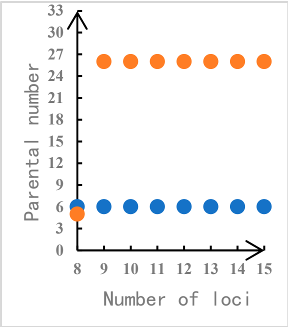

aa) H27

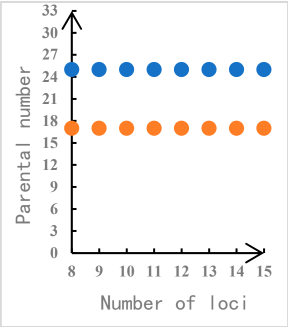

ab) H28

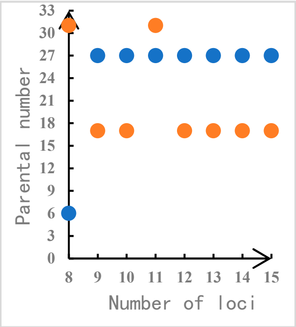

ac) H29

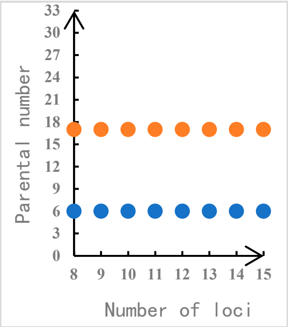

ad) H30

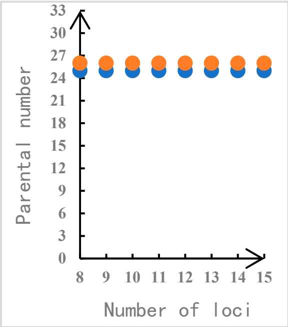

ae) H31

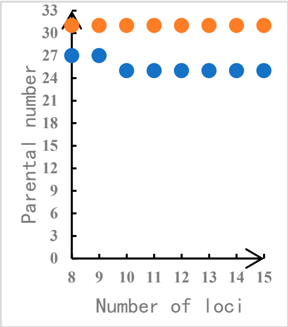

af) H32

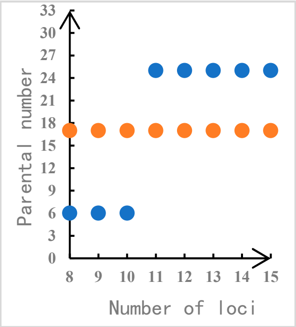

ag) H33

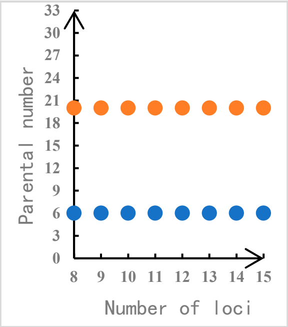

ah) H34

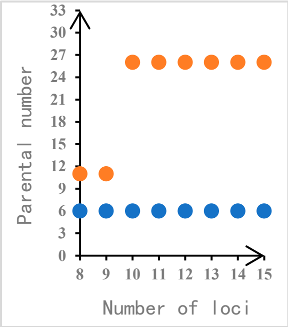

ai) H35

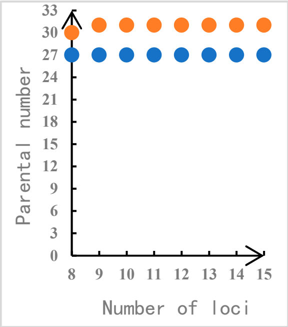

aj) H36

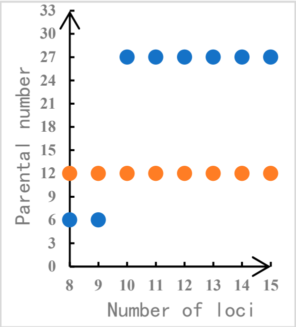

ak) H37

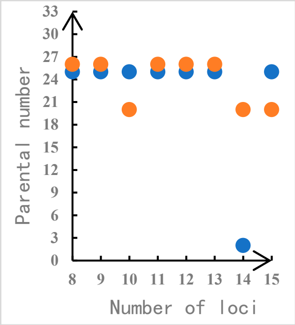

al) H38

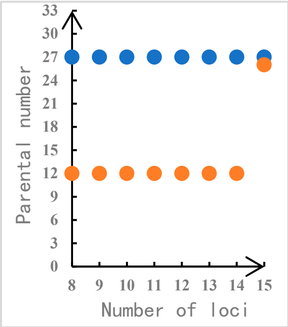

am) H39

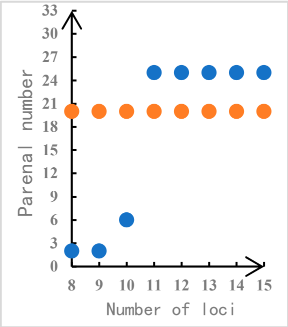

an) H40

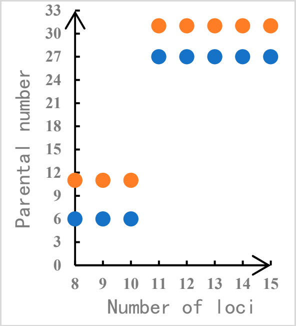

ao) H41

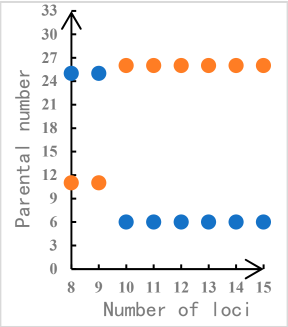

ap) H42

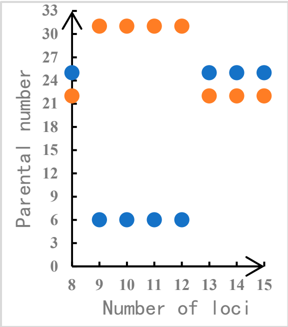

aq) H43

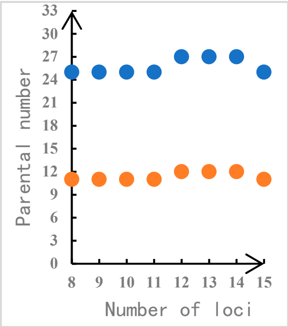

ar) H44

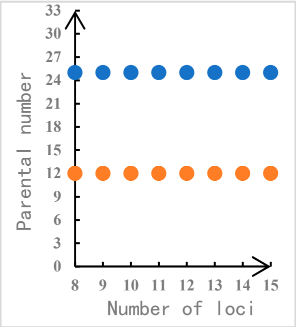

as) H45

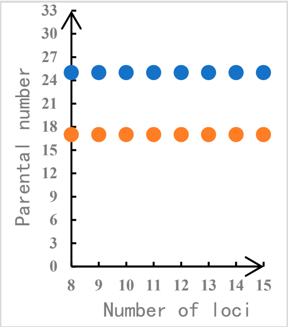

at) H46

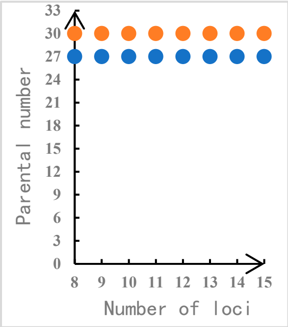

au) H47

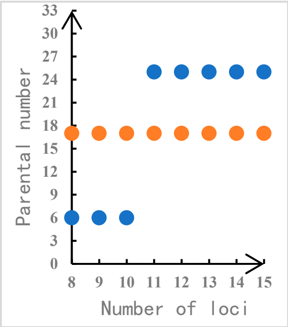

av) H48

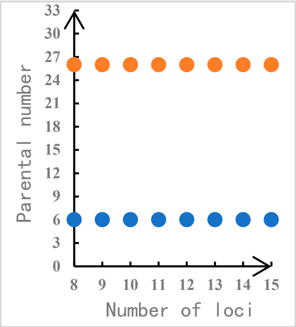

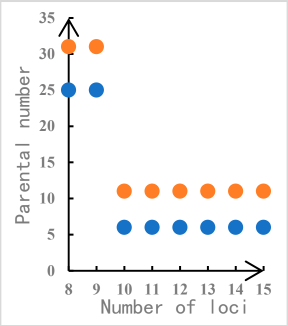

aw) H49 ax) H50

Note: a)–ax) is the number 1–50 individual in the simulated population. Five individuals (H18, H25, H38, H39, and H44) had uncertain parentage, and three individuals (H18, H25, and H39) successfully matched the female parent, and two individuals (H38 and H44) showed potential relationships with to 2 – 3 male parents or female parents.

Supplementary Figure S1. Identification of 50 individuals in the simulated population with different number of loci
